# A Hamster-Derived West Nile Virus Isolate Induces Persistent Renal Infection in Mice

**DOI:** 10.1371/journal.pntd.0002275

**Published:** 2013-06-13

**Authors:** Vandana Saxena, Guorui Xie, Bei Li, Tierra Farris, Thomas Welte, Bin Gong, Paul Boor, Ping Wu, Shao-Jun Tang, Robert Tesh, Tian Wang

**Affiliations:** 1 Department of Microbiology & Immunology, The University of Texas Medical Branch, Galveston, Texas, United States of America; 2 Department of Neuroscience and Cell Biology, The University of Texas Medical Branch, Galveston, Texas, United States of America; 3 Department of Pathology, The University of Texas Medical Branch, Galveston, Texas, United States of America; 4 Center for Biodefense and Emerging Infectious Diseases, The University of Texas Medical Branch, Galveston, Texas, United States of America; 5 Sealy Center for Vaccine Development, The University of Texas Medical Branch, Galveston, Texas, United States of America; Centers for Disease Control and Prevention, United States of America

## Abstract

**Background:**

West Nile virus (WNV) can persist long term in the brain and kidney tissues of humans, non-human primates, and hamsters. In this study, mice were infected with WNV strain H8912, previously cultured from the urine of a persistently infected hamster, to determine its pathogenesis in a murine host.

**Methodology/Principal Findings:**

We found that WNV H8912 was highly attenuated for neuroinvasiveness in mice. Following a systemic infection, viral RNA could be detected quickly in blood and spleen and much later in kidneys. WNV H8912 induced constitutive IL-10 production, upregulation of IFN-β and IL-1β expression, and a specific IgM response on day 10 post-infection. WNV H8912 persisted preferentially in kidneys with mild renal inflammation, and less frequently in spleen for up to 2.5 months post infection. This was concurrent with detectable serum WNV-specific IgM and IgG production. There were also significantly fewer WNV- specific T cells and lower inflammatory responses in kidneys than in spleen. Previous studies have shown that systemic wild-type WNV NY99 infection induced virus persistence preferentially in spleen than in mouse kidneys. Here, we noted that splenocytes of WNV H8912-infected mice produced significantly less IL-10 than those of WNV NY99-infected mice. Finally, WNV H8912 was also attenuated in neurovirulence. Following intracranial inoculation, WNV persisted in the brain at a low frequency, concurrent with neither inflammatory responses nor neuronal damage in the brain.

**Conclusions:**

WNV H8912 is highly attenuated in both neuroinvasiveness and neurovirulence in mice. It induces a low and delayed anti-viral response in mice and preferentially persists in the kidneys.

## Author Summary

West Nile virus (WNV) has been reported to persist long term in the brain and kidney tissues of humans, non-human primates, and hamsters. To define a murine model of persistent WNV renal infection, we characterized infection by WNV H8912, an isolate cultured previously from the urine of a persistently infected hamster. Our findings indicate that WNV strain H8912 is highly attenuated in both neuroinvasiveness and neurovirulence for mice. The virus persisted preferentially in kidneys of the mouse, and less frequently in the spleen and the brain. Moreover, mice infected with WNV H8912 had a delayed induction of IFN- β and IL-1β expression and WNV- specific IgM response, but a constitutive production of serum IL-10. There was a lower proinflammatory response in mouse kidneys when compared to equivalent findings in the spleen. This response may lead to a reduced T cell response in kidneys, which could ultimately contribute to renal-specific WNV persistence. Defining a murine model of WNV persistence by using a well-characterized, hamster-derived WNV urine isolate should provide important insights into understanding the mechanisms of WNV persistence.

## Introduction

West Nile virus (WNV) is a mosquito-borne flavivirus with a positive-sense, single-stranded RNA genome that encodes three structural proteins: the nucleocapsid protein (C), membrane and envelope (E), and seven nonstructural (NS) proteins [1]–[2]. Human infection results from mosquito bites, blood transfusion, organ transplantation, breast feeding and *in utero* or occupational exposure [3]–[6]. About 80% of human infections with WNV are asymptomatic. Among persons with clinical illness, the features of acute illness range from WN fever, to neuroinvasive conditions, including meningitis, encephalitis, acute flaccid paralysis and death [7]–[8]. There is no specific therapeutic agent for treatment of WNV infection, and an approved vaccine for its prevention in humans is not currently available. About 20–50% of WNV convalescent patients have significant long-term morbidity years after their acute illness; symptoms include muscle weakness and pain, fatigue, memory loss, and ataxia [9]–[13]. Although the cause of the persistent sequelae remains undefined, accumulating evidence suggests that persistence of the virus and chronic infection may play a role. Some WNV convalescent patients have been reported to have detectable serum or cerebrospinal fluid (CSF) WNV-specific IgM and - IgA years after the acute infection, which is suggestive of the existence of viral antigens in the periphery or the central nervous system (CNS) of these individuals 14–16. Indeed, WNV antigen or RNA has been detected in the brain or urine of WNV patients ranging from a few months to several years after the initial acute illness [17]–[18].

Persistent WNV infection has also been reported in non-human primates, hamsters and mice [19]–[22]. The first well-documented WNV persistence was reported by Pogodina [20] in non-human primates in 1983, in which infectious virus was mostly detected in the CNS tissues and kidneys for up to 5 ½ months. Experimental infection of hamsters with the WNV NY99 strain induced chronic renal infection and persistent viruria for up to 8 months post- infection, accompanied by moderate renal histopathologic changes [22]–[23]. Siddharthan et al. [21] also demonstrated an active CNS infection and chronic neuropathological lesions in hamsters for up to 100 days after WNV infection. In comparison, infectious virus was cleared from hamster blood and spleen within the first 2 weeks of inoculation [23]–[24]. Following systemic infection of mice with the wild-type WNV, virus persisted preferentially in skin, spinal cord, brain and lymphoid tissues, but the persistence was found less frequently in kidneys [19]. In a recent report, we have demonstrated that infection by a strain of WNV H8912, cultured from urine of a persistently infected hamster, induced a differential proinflammatory cytokine response in mouse macrophage and kidney epithelial cell lines [25]. In the present study, we attempted to further define a murine model of persistent WNV renal infection, using the hamster-derived WNV urine isolate H8912.

## Methods

### Ethics Statement

6- to 10-week-old C57BL/6 (B6) mice were purchased from Jackson Laboratory (Bar Harbor, ME). Groups were age- and sex-matched for each experiment and housed under identical conditions. This study was performed in strict accordance with the recommendations in the Guide for the Care and Use of Laboratory Animals of the National Institutes of Health. All animal experiments were carried out under a protocol approved by the Institutional Animal Care and Use Committee at the University of Texas Medical Branch.

### Infection in mice

Two WNV strains were used: the parental wild-type strain WNV NY385-99 (WNV NY99, [26]), which had been passaged once in African green monkey kidney (Vero) cells and twice in Aedes albopictus (C6/36) cells, and WNV H8912, which was recovered from hamster urine 274 days post-infection after three consecutive passages of a urine isolate from a persistently infected hamster [27]. To evaluate its neuroinvasiveness, mice were inoculated intraperitoneally (i.p.) with 10-fold serial dilutions of WNV H8912, ranging from 10^2^ to 10^6^ PFU. For intracranial (i.c.) infection, anesthetized mice were inoculated with 10^2^, 10^4^, and 10^6^ PFU of WNV H8912 in 50 µl of PBS with 5% gelatin. Infected mice were monitored twice daily for morbidity, including lethargy, anorexia and ataxia.

### Quantitative PCR (Q-PCR) for viral load, cytokine production and leukocyte levels in the CNS

Spleen, kidney and brain tissues were harvested from the WNV-infected mice or controls following perfusion with PBS. For the WNV persistence study, tissues were homogenized and re-suspended in modified Eagle's medium (MEM, Invitrogen, Carlsbad, CA) supplemented with 10% fetal bovine serum (FBS, Sigma, St. Louis, MO). Tissue homogenate was then inoculated into flask cultures of Vero cells three consecutive times (original passage, followed by two blind serial passages harvested at weekly intervals). At the final passage, infected cells were harvested and assayed for WNV RNA by Q-PCR analysis. Non-infected Vero cell samples were used as negative controls. Cells or tissues were resuspended in Trizol (Invitrogen) for RNA extraction. 200 ng RNA was used to synthesize cDNA by reverse transcription (qScript, Quanta Biosciences). The sequences of the primer sets for WNV envelope (*WNVE*), cytokine genes, and genes for leukocytes, such as CD4^+^ and CD8^+^ T cells, neutrophils and monocytes, and PCR reaction conditions were described previously [28]–[31]. The assays were performed in an iCycler (Bio-Rad, Hercules, CA). To normalize the samples, the same amount of cDNA was used in a Q-PCR for *β-actin*. The ratio of the amount of amplified gene compared with the amount of *β-actin c*DNA represented the relative levels in each sample. For *Il-10*, *Tgfβ* and leukocyte genes, results were calculated based on C_t_ values by using the formula 2∧ ^-[C^t^(cytokine or leukocyte gene)-C^t^(*β-actin*)]^ as described in the user manual (SA Biosciences). In the persistence study, samples were considered positive for WNV RNA if the *WNVE* gene expression level was significantly higher than the negative controls.

### Bioplex

Culture supernatant or sera of infected mice were collected for analysis of cytokine production by using a Bio-Plex Pro Mouse Cytokine Assay (Biorad).

### ELISA

Microtiter plates were coated with recombinant WNV-E protein expressed in *Drosophila melanogaster* S2 cells [32] overnight at 4°C at 100 ng/well in coating buffer [0.015 M Na_2_CO_3_, 0.03 M NaHCO_3_, and 0.003 M NaN_3_ (pH 9.6)]. Sera from infected mice were diluted 1/40 in PBS with 2% BSA, added to the duplicate wells, and incubated for 1 h at room temperature. Plates were washed with PBS-Tween (PBS-T). Alkaline phosphatase-conjugated goat anti-mouse IgG or IgM (Sigma) at a dilution of 1/1000 in PBS-T was added for 1 h at room temperature. After washing with PBS-T, color was developed with *p*-nitrophenyl phosphate (Sigma) for 10 min and intensity determined at an absorbance of 405 nm by using a spectrophotometer.

### Kidney leukocyte isolation

Isolation of leukocytes from kidneys was performed by methods described earlier [33]–[34]. Briefly, mice were perfused with PBS, then the kidneys were removed and tissues were mashed through a 70-µm-pore-size strainer in RPMI with 5% FBS. The resulting cell suspensions were centrifuged, and the pellet was resuspended in a 36% Percoll solution (Sigma) and gently overlaid onto 72% Percoll in RPMI, and centrifuged at 1000× *g* for 30 min at room temperature with no brakes. Cells were harvested from the Percoll interface and washed extensively in RPMI with 5% FBS.

### Flow cytometry

Freshly isolated splenocytes and kidney leukocytes were stained with PE-conjugated Pro5 MHC pentamers (H-2Db SSVWNATTA and H-2Kb RSYCYLAT, Proimmune, Oxford, UK) for 10 min at 22°C. WNV- infected BM-DCs were harvested at 48 h post-infection and were stained with antibodies for cell surface markers, including CD80, CD86, MHC II (e-Biosciences). After staining, cells were washed and fixed with 0.5% paraformaldehyde in PBS and acquired by using a C6 Flow Cytometer (Accuri cytometers, Ann Arbor, MI). To measure intracellular cytokine production, splenocytes from WNV-infected mice or controls were isolated and stimulated with WNV-specific NS3 and E peptides (RRWCFDGPRTNTILE and PVGRLVTVNPFVSVA, respectively [35]) for CD4 T cells, or WNV-specific NS4B and E peptides (SSVWNATTA and IALTFLAV, respectively [36]–[37]) for CD8 T cells for 5 h at 37°C. Golgi-plug (BD Biosciences) was added at the beginning of stimulation. Cells were harvested, stained with antibodies for CD4 or CD8, fixed in 2% paraformaldehyde, and permeabilized with 0.5% saponin before adding PE-conjugated anti-IFN-γ, or control PE-conjugated rat IgG1 (eBiosciences, San Diego, CA).

### Histologic examination of tissues

Anesthetized mice were perfused with 30 ml of ice cold PBS. Brains and kidneys were removed and fixed in 4% paraformaldehyde. Subsequently, specimens were transferred to 70% ethanol and processed for histopathologic examination. Five-micron paraffin sections were prepared for staining with hematoxylin & eosin, in the case of kidneys, six sections were made for each mouse. Stained sections were examined for lesions by a pathologist, who was blinded to the origin of the samples. As a lesion was defined, its incidence was scored based on presence or absence in a single section, thus allowing for determination of % incidence in the histologic material.

### Cell culture and virus infection

Bone-marrow (BM)- derived dendritic cells (DCs) were generated as described previously [38]. Briefly, BM cells from B6 mice were isolated and cultured for 6 days in RPMI-1640 supplemented with granulocyte-macrophage-colony stimulating factor, and interleukin-4 (Peprotech) to generate myeloid DC. Day 6-cultured DCs were infected with WNV NY99 or WNV H8912 strain at a multiplicity of infection (MOI) of 0.2. Primary cortical cultures from mouse embryos of either sex were prepared as described previously [39]. Briefly, cortices were dissected from B6 mouse embryos (E18) and washed in Hanks' balanced salt solution (HBSS, Invitrogen). Meninges and excess white matter were removed. Cortices were chopped into small tissue blocks (0.5–1 mm^3^) and transferred to a sterile tube containing HBSS. Cortical tissues were treated with 0.25% trypsin (Sigma) for 8 min at 37°C, and dissociated to single cell suspensions, followed by centrifugation at 120× g for 5 min. The cell pellets were re-suspended in Dulbecco's Modified Eagle Media (DMEM, Invitrogen) containing 10% FBS and 1% penicillin-streptomycin. Cells were inoculated into culture plates coated with 20 µg/ml poly-D-lysine (PDL, Sigma) in 0.1 M borate buffer (PH 8.5, 50 mM H_3_BO_3_, 12.5 mM Na_2_B_4_O_7_(10H_2_O)). Two hours later, DMEM was replaced with Neurobasal Medium (Invitrogen) supplemented with 2% B27 (Invitrogen), 1% penicillin-streptomycin, and 0.5 mM L-glutamine. Cells were cultured at 37°C and one-third of the medium was replenished every 3 days until 10 days *in vitro*. For WNV infection, 0.3×10^6^ cells were grown on the coverslips in a 24-well tissue culture plate and infected with WNV NY99 or WNV H8912 at a MOI of 0.003. At indicated time points post-infection, supernatants were collected and measured for viral titer by plaque assay. Cells were also harvested and resuspended in Trizol (Invitrogen) for RNA extraction. Viral load was determined by Q-PCR assay.

### Plaque assay

Vero cells were seeded in 6-well plates in DMEM (Invitrogen) supplemented with 10% FBS 24 h before infection. Serial dilutions of cell culture supernatants of both NY99 and H8912 infected cells were added and incubated for 1 h. Subsequently, DMEM containing 2% FBS and 1% low-melting-point agarose were added and the plates were incubated for 3 days. A second overlay of 4 ml 1% agarose-medium containing 0.01% neutral red (Sigma) was added to visualize plaques. Virus concentrations were determined as PFU/ml.

### Statistical analysis

Data analysis was performed by using Prism software (Graph-Pad) statistical analysis. Values for viral burden, plaque assay, and cytokine production experiments were presented as means ± SEM. *P* values of these experiments were calculated with a non-paired Student's t test. Statistical significance was accepted at *P*<0.05.

## Results

### WNV H8912 isolate was highly attenuated in neuroinvasiveness in mice

**Figure 1:**
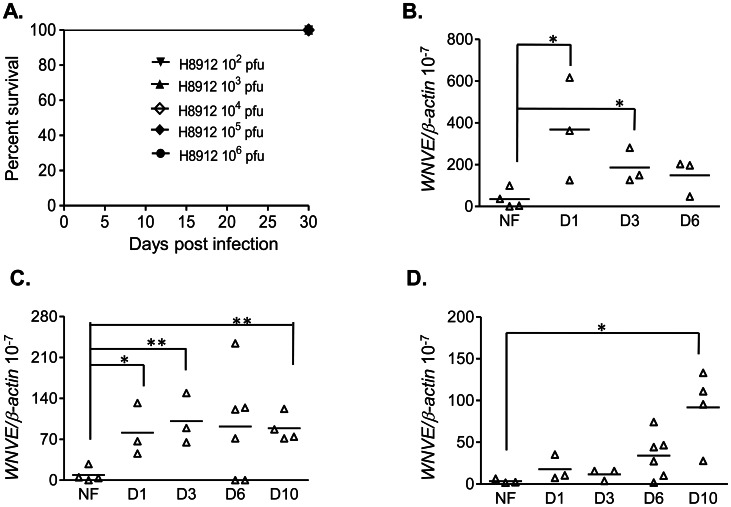
WNV H8912 infection in mice. *A.* Mice were injected with a 10-fold serial dilution of WNV H8912 and monitored twice daily (n = 3 per group). *B–D.* Viral load in blood (*B*), spleen (*C*) and kidneys (*D*) was determined by using Q-PCR at the indicated time points post-infection. Data are presented as means ± SEM, n = 3–6. * *P*<0.05 or **P<0.01 compared to non-infected (NF) mice.

WNV H8912 was originally isolated from urine of a WNV persistently infected hamster. It has been shown to be highly attenuated in neuroinvasiveness and to induce chronic renal infection in hamsters [27]. To characterize WNV H8912 infection in a murine model, we infected B6 mice i.p. with 10-fold serial dilutions of 10^2^ to 10^6^ PFU of WNV H8912. Infected mice were monitored twice daily for morbidity for over a month. All mice survived infection (**Fig. 1A**); and less than 20% of the mice infected with the highest dose (10^6^ PFU) of WNV H8912 developed mild disease symptoms (ruffeled fur, irritability, etc). We also measured viral load in blood, spleen and kidneys in B6 mice following an i.p. inoculation of 500 PFU of WNV H8912. At days 1, 3, 6 and 10 post-infection, tissues were collected, and viral load was measured by a Q-PCR assay. As shown in **Fig. 1B**, viremia increased quickly by day 1 and declined on day 6 post-infection compared to equivalent findings in naïve mice (*P*<0.05). Viral load in the spleen was also increased on day 1 and continued to be detectable on day 10 post-infection (**Fig. 1C**, *P*<0.05 or *P*<0.01). In contrast, viral load in kidneys was not detectable until on day 10 post-infection (**Fig. 1D**, *P*<0.05). These results indicate that WNV H8912 has a highly reduced neuroinvasiveness in mice, and that its replication in blood, spleen and kidneys showed differential kinetics.

### Innate cytokine and antibody production in WNV H8912-infected mice

**Figure 2:**
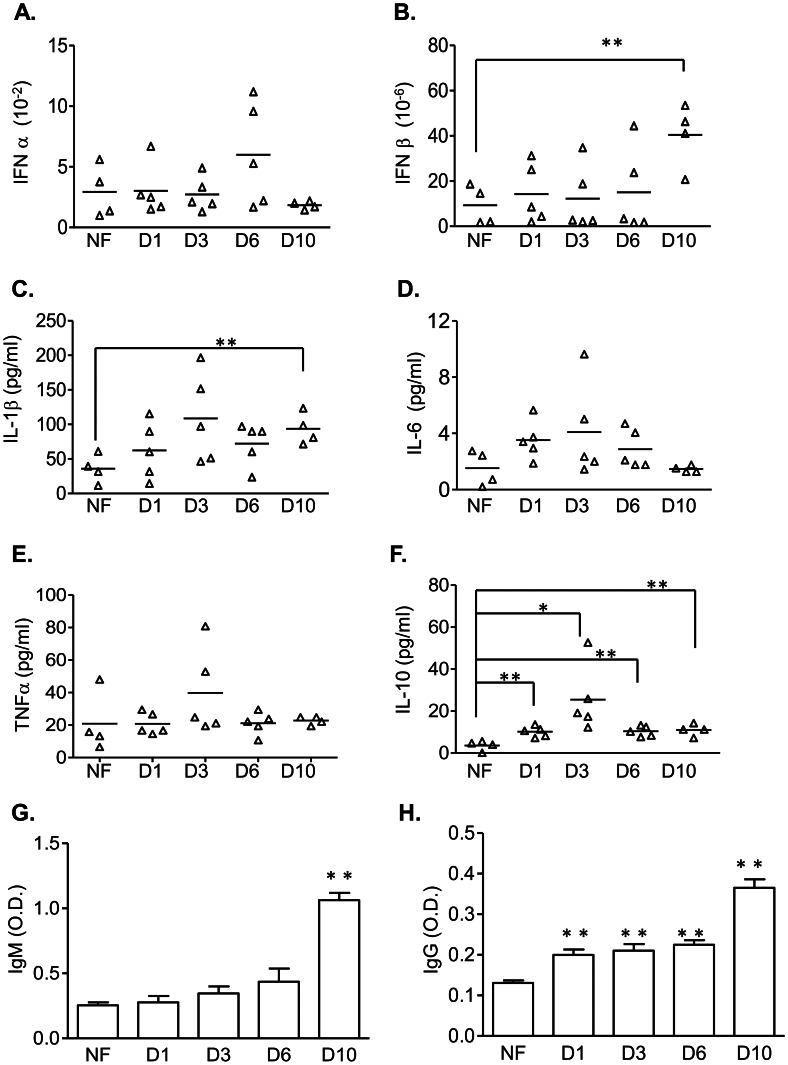
Innate cytokine and antibody production in WNV H8912- infected mice. *A–F.* Cytokine levels in blood at the indicated time points post-infection were determined by using Q-PCR or Bioplex. *G–H.* Sera were collected from non-infected (NF) mice or those infected with WNV H8912 at day 1 (D1), day 3 (D3), day 6 (D6) and day 10 (D10) post-infection. The development of WNV-specific IgM (*G*) or IgG (*H*) antibodies was determined after incubating sera with absorbed purified r-WNV-E protein. Data are presented as means ± SEM, n = 4–6. * *P*<0.05 or **P<0.01 compared to the NF group.

Innate cytokine responses, including type 1 interferon (IFN)s, proinflammatory cytokines and regulatory cytokines, play an important role in protection and pathogenesis during wild-type WNV NY99 infection [29], [40]–[42]. To examine cytokine production, we infected mice i.p. with 500 PFU of WNV H8912. Blood was collected at days 1, 3, 6 and 10 post-infection for cytokine measurement by Q-PCR and Bioplex. Compared to the samples from non-infected mice, IFN-α gene expression was not changed at any of the time points examined (**Fig. 2A**, *P*>0.05). There was a 3–4 fold induction in IFN-β gene expression on day 10 post infection (**Fig. 2B**, *P*<0.01). Higher interleukin (IL)-1β production was also detected in serum on day 10 post infection (**Fig. 2C**, *P*<0.01). No changes were noted in the expression of the two genes at earlier time points nor in the production of other proinflammatory cytokines, including IL-6 and tumor necrosis factor (TNF)-α (**Figs. 2B–2E**, *P*>0.05). We found that WNV H8912 constitutively induced serum IL-10 levels compared to those in naïve mice (**Fig. 2F**, *P*<0.01 or *P*<0.05). B cell-mediated humoral immune responses are critical for host defense against disseminated infection by WNV [43]–[45]. In particular, induction of a specific, neutralizing IgM response early in the infection limits viremia and dissemination into the CNS and protects the host against lethal wild-type WNV infection [46]. Interestingly, WNV H8912-infected mice had no detectable serum WNV-specific IgM until at day 10 post-infection (**Fig. 2G**, *P*<0.01). In comparison, there was a low WNV-specific IgG production at days 1 to 6 post-infection and the titer was further increased at day 10 (**Fig. 2H**, *P*<0.01). Together, these results suggest to us that WNV H8912- infected mice had a delayed induction of IFN-β and IL-1β expression and IgM response, but a constitutive production of serum IL-10.

### WNV H8912 persisted preferentially in mouse kidneys following a systemic infection

**Figure 3:**
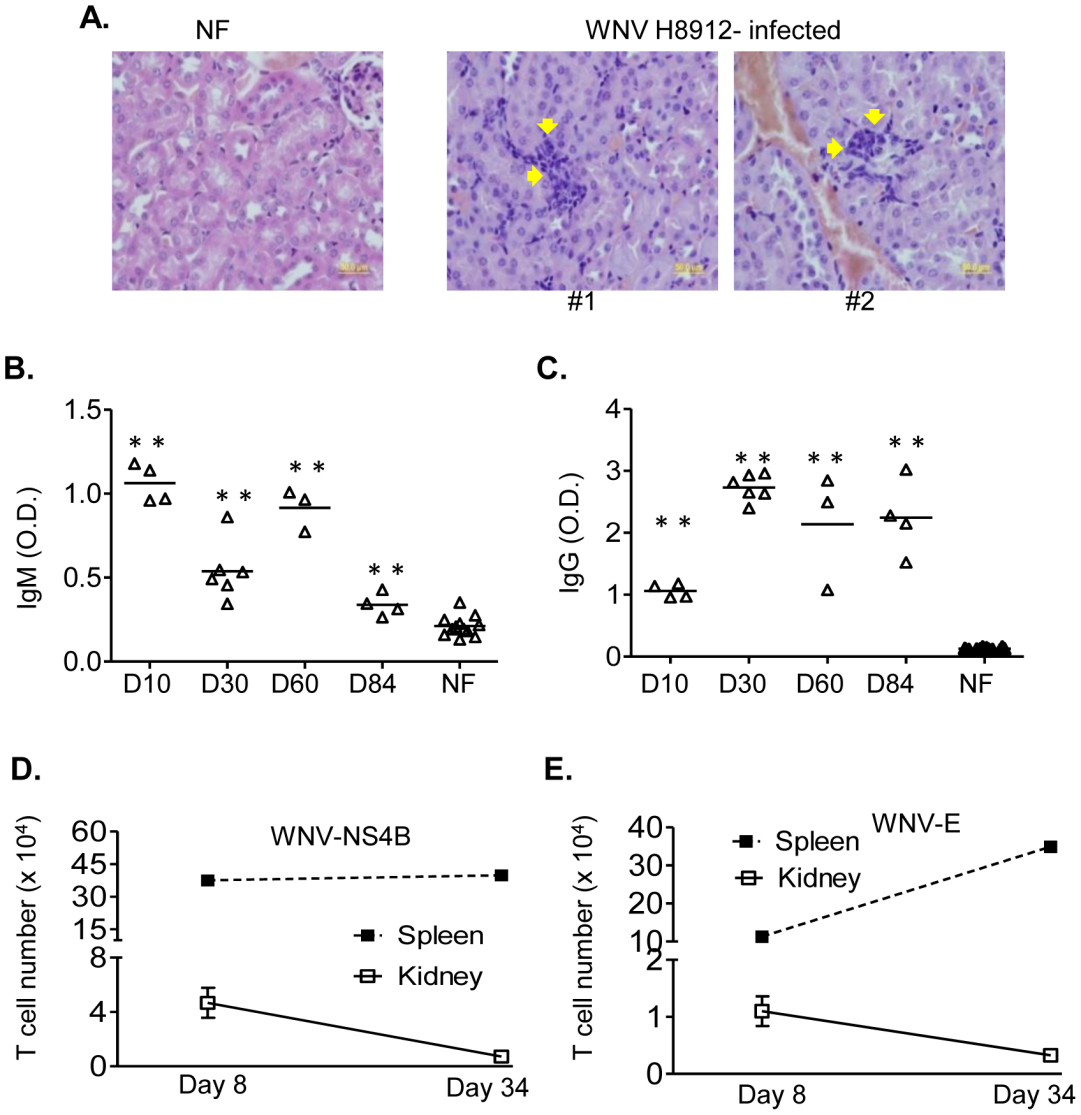
Kidney pathology and immune responses in WNV H8912 -infected mice. *A.* Hematoxylin and eosin staining of control non-infected (NF) mouse and two WNV H8912-infected mice kidney sections at day 84 post-infection; arrows in infected mouse kidney indicate focal interstitial lymphocytic collection. *B–C.* Sera were collected from NF and mice that were infected with WNV H8912 at day 10 (D10), day 30 (D30), day 60 (D60) and day 84 (D84) post-infection. The development of WNV-specific IgM (*B*) or IgG (*C*) antibodies was determined after incubating sera with absorbed purified r-WNV-E protein. Data are presented as means ± SEM, n = 4–12. **P<0.01 compared to NF mice. *D–E.* Splenocytes and kidney leukocytes were harvested at indicated time points post-infection and stained for CD8 and WNV- NS4B or WNV -E epitopes. The total numbers of WNV specific T cells were shown.

In a previous report, the hamster-passaged WNV urine isolates, including WNV H8912 were shown to induce persistent renal infection in hamsters following a systemic infection [27]. Here, we wondered whether WNV H8912 would induce persistent infection in mice. At days 30, 60 and 84 post-infection, spleens and kidneys were harvested from infected mice, and the tissue homogenate was inoculated into Vero cells consecutively for three times before detection of WNV RNA by Q- PCR assay. WNV RNA was equally detected in both tissues at day 30 but at a higher frequency in kidneys than in spleen tissues at days 60 and 84 post-infection (**Table 1**). Furthermore, in mice at day 84 post-infection, pathologic examination revealed a sporadic, focal chronic inflammation consisting of clusters of lymphocytes in the intertubular interstitium of all four kidney samples of experimental mice that were detected as positive for WNV RNA (**Fig. 3A** **, right panel**). The incidence of the inflammatory lesion varied from 17% to 83% (number of sections with lesion present per 6 total sections/mouse) with a mean observance of 46%±14%. No such inflammatory lesions were observed in equivalent numbers of kidney sections of control mice (**Fig. 3A** **, left panel**). Interestingly, we noted that there was a detectable serum WNV-specific IgM response at all these time points, though it dropped at days 30 and 84 post-infection compared to that of day 10 (**Fig. 3B**, *P*<0.01). In comparison, WNV-specific IgG production was increased at days 30 and 60 post infection compared to that at day 10 (**Fig. 3C**, *P*<0.01). Both WNV-E- and NS4B-specific CD8^+^ T cells dominated during wild-type WNV NY99 strain infection [36]–[37]. In WNV H8912-infected mice, we found that the number of these two T cell populations in the splenic tissues were either maintained or increased at day 34 post-infection compared to those on day 8 post-infection (**Figs. 3D & 3E**), whereas the number of both T cell populations was significantly lower in kidney tissues and was even reduced at the late stage of infection (day 34) compared to those at day 8 (**Figs. 3D & 3E**). To understand the underlying mechanism of WNV persistence, we measured and compared the expression of innate cytokines in both tissues. Our results showed that IL-6 expression levels in the spleen were induced during the early stage of infection (days 1 to 10 post-infection), but were reduced at days 30 and 60. In comparison, no induction of IL-6 expression was observed in the kidneys of WNV H8912-infected mice (**Fig. S1A**). The TNF-α expression level was slightly increased in spleens throughout the course of infection, whereas it was decreased in kidneys of H8912-infected mice except at day 6 post-infection (**Fig. S1B**). Further, we did not note any significant differences in the expression of regulatory cytokines, such as TGF-β and IL-10, in these two tissues during WNV H8912 infection (**Figs. S1C & S1D**). These results suggest that WNV H8912 induces a persistent infection preferentially in mouse kidneys rather than in the spleen, possibly due to the differential proinflammatory cytokine expression and T cell responses in these two tissues.

**Table 1:** Persistent WNV infection in spleen and kidneys.

Days Post-infection	Spleen (QPCR +/total)	Kidney (QPCR +/total)
30	6/7	6/7
60	4/6	6/6
84	1/4	4/4

Spleen and kidney tissues were harvested from WNV-infected mice at indicated time points post-infection. Tissue homogenates were passaged serially in Vero cells for three times. Inoculated cells were harvested and assayed for viral RNA by Q-PCR analysis. Four to seven of non-infected Vero cell samples were used each time as negative controls. Samples were considered to be positive for WNV RNA if the *WNVE* gene expression level was higher than the negative controls (*P*<0.05 or *P*<0.01 as determined by a non-paired Student's t test).

**Figure 4:**
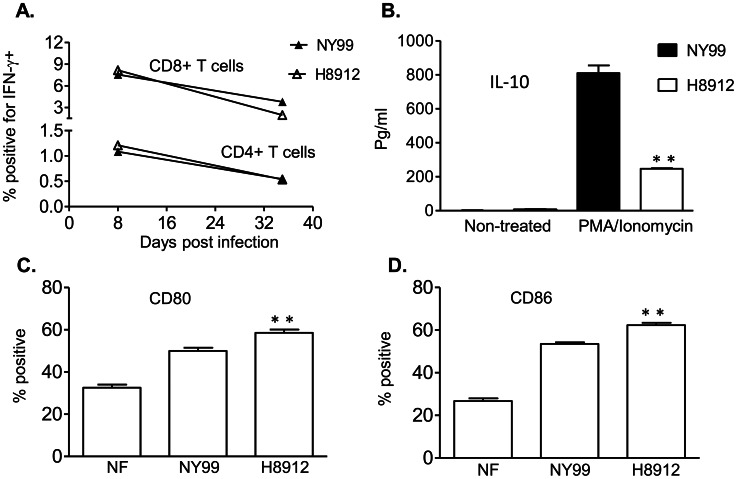
Splenic T cell responses and DC maturation during WNV NY99 or H8912 infection. *A–B.* Splenocytes of WNV NY99 or H8912-infected mice at day 8 and day 35 post-primary infection were cultured *ex vivo* with WNV peptides for 5 h (*A*) or with PMA plus ionomycin (*B*) for 24 h. *A.* Cells were harvested and stained for IFN-γ, and CD4 or CD8. *B.* Cell culture supernatant was collected and IL-10 was measured by Bioplex. *C–D.* DC maturation in WNV-infected BM-DCs. The percentages of CD80^+^ (*C*) and CD86^+^ (*D*) on BM-DCs were shown. Data are presented as means ± SEM, *n* = 3 or 4. **P<0.01 for WNV NY99 vs. WNV H8912.

A previous report shows that systemic wild-type WNV NY99 infection induced virus persistence preferentially in the spleen than in mouse kidneys [19]. We next compared splenic CD4^+^ and CD8^+^ T cell responses following WNV H8912 or NY99 infection. We noted CD4^+^ and CD8^+^ T cells of WNV H8912 or NY99-infected mice produced similar levels of IFN-γ upon *ex vivo* stimulation with WNV peptides on days 8 and 38 post-infection (**Fig. 4A**). Nevertheless, splenocytes of WNV H8912-infected mice induced significantly less IL-10 production compared to those of WNV NY99-infected mice upon *ex vivo* stimulation with PMA and ionomycin (**Fig. 4B**, *P*<0.01). DCs represent the most important antigen- presenting cells exhibiting the unique capacity to initiate primary T cell responses and are permissive to WNV infection. At day 2 post-infection, we noted 18–19% increase on the percentage of CD80 and CD86 expression on WNV H8912-infected BMDCs when compared to WNV NY99-infected cells respectively (**Figs. 4C & 4D**, *P*<0.01). Thus, a decreased IL-10 production by WNV H8912-infected splenocytes may contribute to the lower frequency of virus persistence in spleen compared to that of WNV NY99-infected mice.

### WNV 8912 is also highly attenuated in neurovirulence. WNV persistence was found at a low frequency in the brain following an i.c. infection

**Figure 5:**
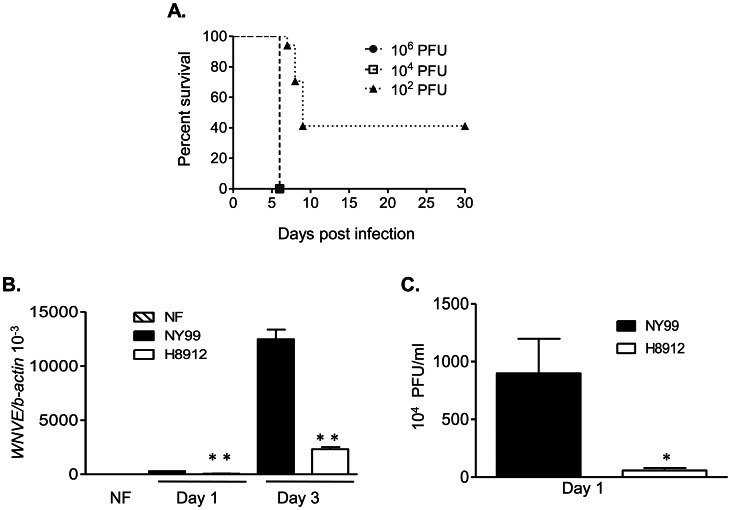
WNV H8912 exhibited a significantly reduced neurovirulence. *A.* Mice were injected i.c. with different doses of WNV H8912 and monitored twice daily. *B–C.* Mouse primary cortical neuron/glia cultures were infected with WNV H8912 and its wild-type controls WNV NY99. Viral load at indicated time points post-infection was determined by Q-PCR assay (*B*) or by plaque assay (*C*). Data are presented as means ± SEM, n = 3. * *P*<0.05 or **P<0.01 compared to WNV NY99-infected cells.

**Figure 6:**
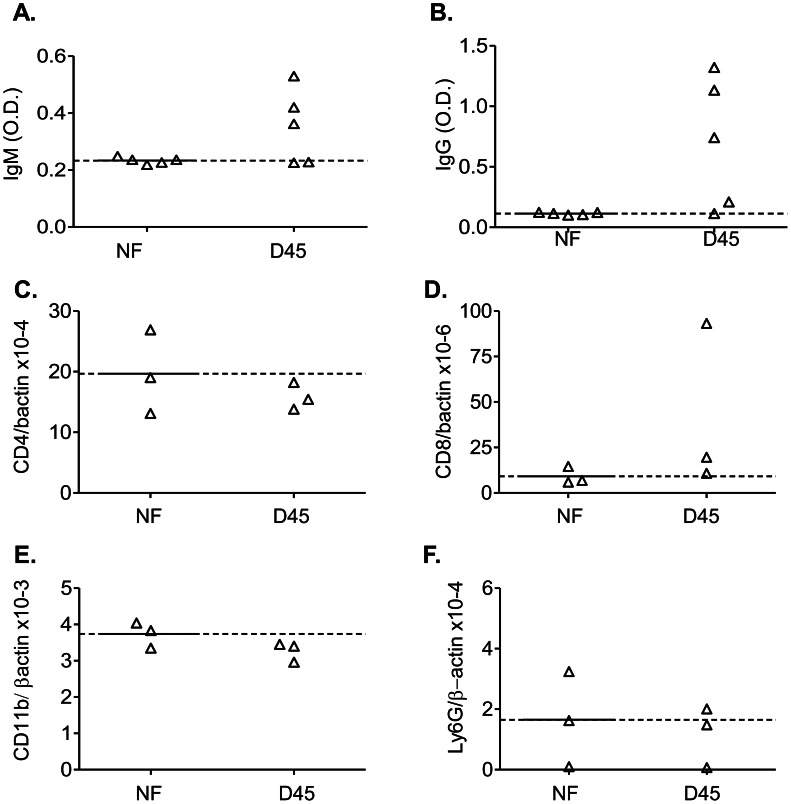
Antibody production and brain leukocyte infiltration at day 45 post- infection. *A–B.* Sera were collected from non-infected (NF) or mice which were intracranially (i. c.) infected with WNV H8912 at day 45 (D45) post-infection. The development of WNV-specific IgM (*A*) or IgG (*B*) antibodies was determined. *C–F.* Levels of CD4^+^ T cell (*C*), CD8^+^ T cell (*D*), monocyte (CD11b, *E*) and neutrophil (Ly6G, *F*) expression in the three WNV-infected mice brains were measured by Q-PCR assay at day 45 post-infection. The *y*-axis depicts the ratio of the amplified *CD4*, *CD8, CD11b or Ly6G* cDNA to *β-actin* cDNA of each sample. The dotted line represents the mean of NF samples.

To determine the neurovirulence of WNV H8912, we infected mice i.c. with 10^2^, 10^4^ and 10^6^ PFU of WNV H8912. Infected mice were monitored twice daily for morbidity and mortality for over a month. All mice inoculated with 10^4^ and 10^6^ PFU of WNV H8912 infection died; while 41% of mice inoculated i.c. with 10^2^ PFU of WNV H8912 survived (**Fig. 5A**). To further study its neurovirulence, we infected mouse primary cortical neuron/glia cultures with WNV H8912 and its wild-type control WNV NY99. At days 1 and 3 post-infection, the viral load in WNV H8912-infected cells was about 75%–80% lower than that in WNV NY99-infected cells as measured by Q-PCR analysis (**Fig. 5B**, *P*<0.01). Plaque assay results also showed a 94% decrease in viral titers of WNV H8912- infected cells compared to that of WNV NY99-infected (**Fig. 5C**, *P*<0.05) on day 1 post-infection. We next studied WNV persistence in the brains of mice that survived the 10^2^ PFU of WNV H8912 i. c. infection. At day 45 post-infection, WNV RNA was only detected by Q-PCR assay in one of the five surviving mice (20%, data not shown). Among the surviving mice, 60% had a detectable serum WNV-specific IgM response; whereas 80% of them were positive for WNV-specific IgG (**Figs. 6A and 6B**). To determine if WNV persistence in the brain was accompanied by inflammatory responses, we studied brain pathology. On day 45 post-infection, we did not detect significant histologic evidence of inflammation or neuronal damage in the cortex and olfactory bulb regions of the brain of surviving mice, when compared to equivalent tissues in non-infected controls (**Figs. S2A & S2B**). In further phenotype analyses of the brain leukocytes by Q-PCR assay, no significant infiltration of either CD4^+^ T cells (**Fig. 6C**), monocytes (CD11b^+^, **Fig. 6E**), or neutrophils (Ly6G^+^, **Fig. 6F**) was observed in the brains of the surviving mice. Nevertheless, we noted one of the surviving mice- which was also positive for viral RNA, had more CD8^+^ T cell infiltrates in the brain (**Fig. 6D**) at day 45 post-infection than non-infected controls. Collectively, these results suggest that WNV H8912 is highly reduced in neurovirulence. It appears to induce WNV persistence much less frequently in the brain than in the kidneys with no histopathologic changes in the CNS.

## Discussion

WNV H8912, a viral isolate recovered from urine of persistently infected hamster, was attenuated in neuroinvasiveness but capable of producing chronic renal infection in hamsters [27]. In this study, we found that WNV H8912 was also highly attenuated in neuroinvasiveness and neurovirulence for mice. Following a systemic infection with WNV H8912, viral RNA was detected more frequently in kidneys than in spleen by a Q-PCR assay. This was concurrent with a focal chronic inflammation response in the inter-tubular interstitium of kidney samples found to be positive for WNV RNA on day 84-post-infection. In comparison, WNV persisted at a lower frequency in the brain following i.c. inoculation with no significant changes in the CNS. In summary, our results suggest that WNV H8912 was highly attenuated and induced a tissue-specific, persistent renal infection in mice.

B cell-mediated humoral immune responses are critical in host defense against disseminated infection by WNV [43]–[45]. Here, we found that WNV H8912 induced a delayed serum WNV-specific IgM and a low WNV-specific IgG production in the early stages of infection, which may contribute to its persistence in the peripheral tissues. Moreover, the WNV-specific IgM response remained detectable in the later stages of infection and decreased only slightly at days 30 and 84 post-infection, compared to that on day 10. WNV-specific IgG production was enhanced at days 30 and 60 post-infection, compared to that in the early stage of infection. The detection of WNV-specific IgM and IgA in sera of WNV convalescent patients years after having the acute infection has been reported and appears to be correlated with the persistence of viral RNA in the kidneys, urine or the CNS of these patients [14]–[16]. Thus, these results further support that WNV H8912 induces persistent infection in mouse peripheral tissues. T cells are important for host survival following wild-type WNV infection and contribute to a long-lasting protective immunity [47]–[48]. Elevated levels of IL-10 during chronic viral infections are known to contribute to diminished T cell activity and the failure to control viral infection [49]–[50]. Interestingly, sera IL-10 levels in WNV H8912-infected mice were constitutively high within the first two weeks of infection. There were also delayed anti-viral responses of IFNs and IL-1β in these mice. Collectively, these factors may potentiate persistent viral infection in mouse peripheral tissues following systemic WNV H8912 infection.

Systemic wild-type WNV NY99 infection induced virus persistence more frequently in spleen than in mouse kidneys [19]. In this study, we found WNV H8912 induced virus persistence preferentially in kidneys following an i.p. infection. Multiple virus and host determinants could contribute to a tissue –specific WNV persistence in mice. First, our recent work showed that WNV H8912 had a significantly reduced replication rate in mouse kidney epithelial cells compared to wild-type WNV strain [25], which indicate a greater tissue tropism is not involved in WNV H8912-induced renal-specific persistence. Differential immune responses have been reported to contribute to tissue-specific viral persistence. For example, a Treg-mediated suppression of CD8^+^ T cell response occurred specifically in the spleen but not in the liver during chronic Friend virus infection. This resulted in a 10-fold less viral load in the latter tissue [51]. Here, we next measured proinflammatory cytokine responses in spleen and kidney tissues and found there were lower levels of IL-6 and TNF-α expression in the kidneys of WNV H8912-infected mice compared to spleen tissues. This is consistent with our recent findings, in which WNV H8912 infection in mouse kidney epithelial cells did not induce IL-6 and TNF-α expression [25]. DC maturation is an innate response that leads to adaptive immunity to foreign antigens [52]–[53]. Proinflammatory cytokines are known to promote this process [54]–[55]. We found significantly less WNV-specific CD8^+^ T cells in WNV persistently infected kidneys than in the spleen tissues throughout the infection. Thus, a lower inflammatory response in the kidneys may lead to reduced WNV specific T cell infiltrates and ultimately to a preferential WNV persistence in mouse kidneys. Lastly, our results also showed that splenocytes of WNV H8912-infected mice produced much less IL-10 than those of wild-type strain-infected mice. There were higher levels of expression of DC maturation markers on WNV H8912-infected BMDCs than wild-type WNV –infected cells. In conclusion, differential proinflammatory cytokine and IL-10 responses in spleen and kidney tissues lead to tissue-specific virus persistence during systemic WNV infection. WNV H8912 is highly attenuated in mice. It induced a low frequency of virus persistence in the CNS following i.c. inoculation, concurrent with no significant changes in the cortex and olfactory bulb regions of the brain of surviving mice. Neurons and microglias are the two major cell types permissive to WNV infection [29], [56]. Our results showed that WNV H8912 had a significantly lower titer in mouse primary cortical neuron/glia cultures compared to wild-type WNV strain, which suggests WNV H8912 does not have a tissue tropism in the CNS. While the underlying mechanisms by which WNV H8912-induced a low frequency of viral persistence are under investigation, these results further support that WNV H8912 induces a tissue-specific renal persistence.

WNV encephalitis (neuroinvasive disease) has been a serious public health concern in North America for more than a decade. Neither treatment nor human vaccines are available. In recent years, some WNV convalescent patients have been reported to have persistent sequelae, which occurred 6 to 12 months after the acute infection [9], [11]. Murray et al. [18] detected the presence of WNV RNA in the urine of patients convalescent from WNV neuroinvasive disease for up to 1–7 years after their initial infection. Among these WNV RNA-positive patients, 80% reported chronic neurological symptoms, while 20% had renal failure [18]. These observations raise the possibility that persistent infection is associated with WNV-induced chronic diseases. Persistent WNV infection was detected in the hamster kidneys and brain, accompanied by neurological sequelae [21]–[23]; this association is similar to the clinical findings in some WNV convalescent patients having long-term morbidity. Compared to the parent wild-type NY99 strain, full genome sequencing reveals conserved genetic mutations in the coding and non-coding regions of the viral genome of all WNV isolates recovered from urine of persistently infected hamsters [27], including WNV H8912, indicating an association of genetic mutations with WNV persistence renal tropism. Thus, hamster model would likely serve as a suitable *in vivo* model to further investigate the underlying immune mechanisms of WNV persistence. Nevertheless, there is an obstacle to fully investigating immunity to chronic WNV infection in the hamster model, due to limited availability of hamster reagents. Mice are easier to work with, amenable to immunological manipulation and are relatively inexpensive. Systemic wild-type WNV NY99 infection in mice induced virus persistence preferentially in spleen tissues than in kidneys [19]. Our study has now shown that the hamster-derived WNV urine isolate H8912 induced persistent infection in mouse kidneys, accompanied by focal renal histological inflammatory changes for up to a few months post infection, which is similar to the observations in WNV convalescent patients having long-term morbidity. Thus, defining a murine model of WNV persistence by using a well-characterized, hamster-derived WNV urine isolate should provide important insights into the mechanisms of WNV CNS persistence and its associated neurological sequelae. This information may enable us to define the elements of the immune response that fail or are insufficient to mediate WNV clearance in the infected animals and ultimately help us to set therapeutic goals to modulate immune functions pharmacologically and create vaccines which could induce robust T cell memory responses.

## Supporting Information

Figure S1 **Cytokine levels in spleen and kidney tissues in WNV H8912- infected mice.** IL-6 (*A*), TNF-β (*B*), TGF-β (*C*) and IL-10 (*D*) gene expression in the spleen and kidney tissues at the indicated time points post infection were determined by using Q-PCR. The fold of increase compared to that of the mock group is shown. Data are presented as means ± SEM, n = 3–6.
(TIF)

Figure S2 **Brain pathology at day 45 post-WNV H8912 infection.** Hematoxylin and eosin staining of the cortex (*A*) and olfactory bulb (*B*) regions of control non-infected (NF) and WNV H8912-infected mouse (IF) brain sections at day 45 post- i.c. infection.
(TIF)
