# Supplementary figures and images for: A Hamster-Derived West Nile Virus Isolate Induces Persistent Renal Infection in Mice
Source: PLoS Negl Trop Dis. 2013 Jun 13;7(6):e2275. doi: 10.1371/journal.pntd.0002275 (PMC3681636; doi:10.1371/journal.pntd.0002275)

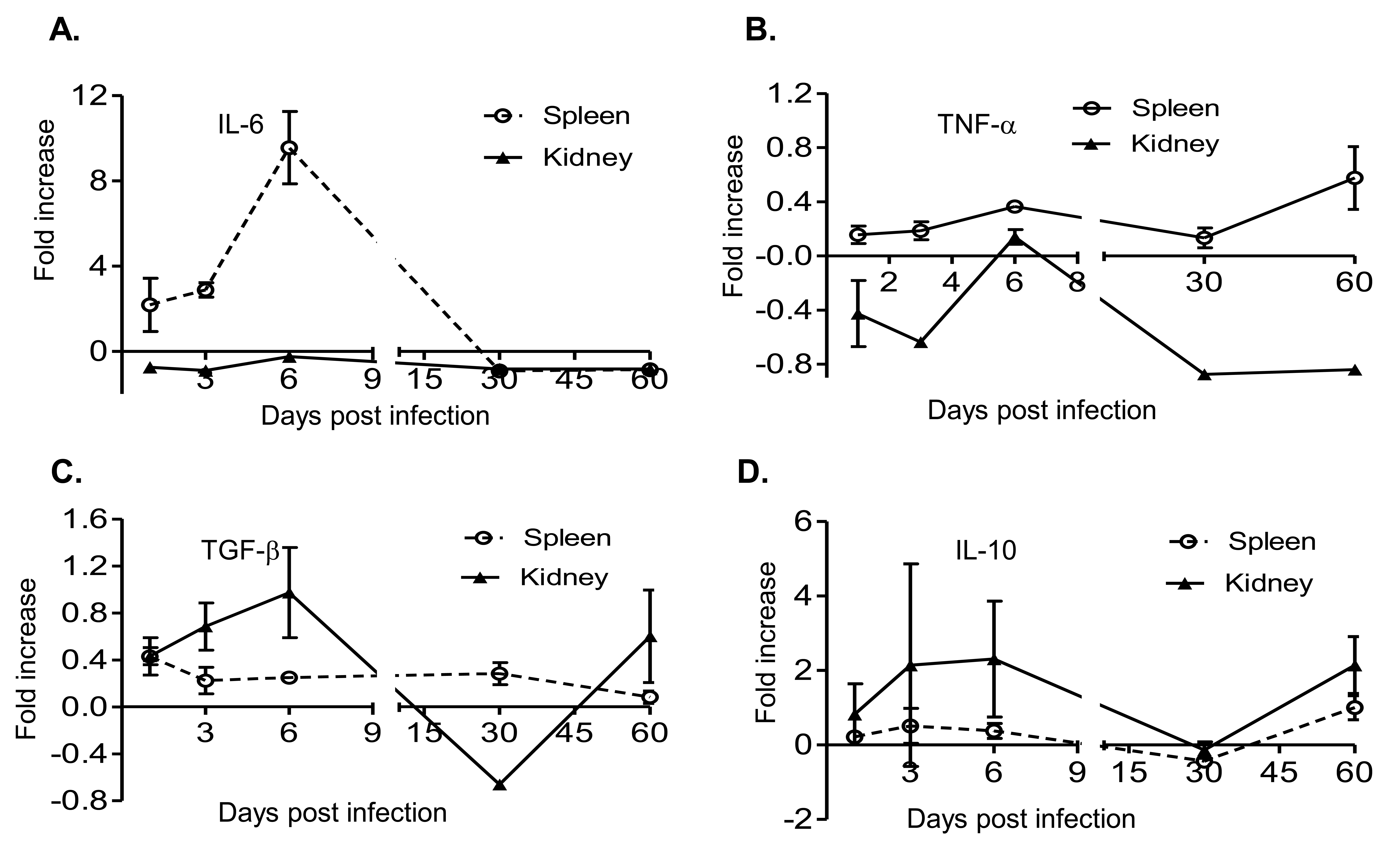

Supplement: Figure S1 — Cytokine levels in spleen and kidney tissues in WNV H8912- infected mice. IL-6 (A), TNF-β (B), TGF-β (C) and IL-10 (D) gene expression in the spleen and kidney tissues at the indicated time points post infection were determined by using Q-PCR. The fold of increase compared to that of the mock group is shown. Data are presented as means ± SEM, n = 3–6. (TIF) [file pntd.0002275.s001.tif]

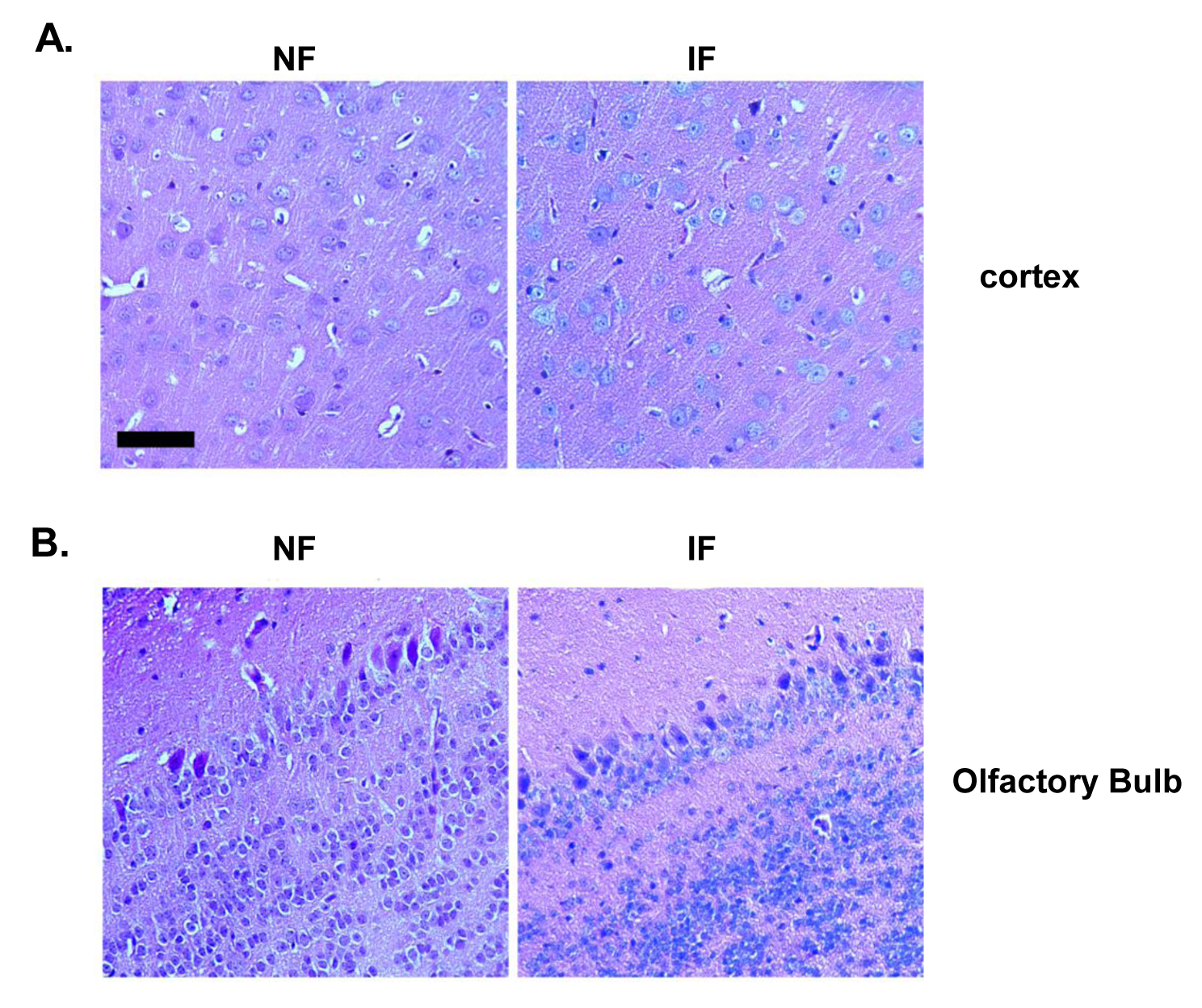

Supplement: Figure S2 — Brain pathology at day 45 post-WNV H8912 infection. Hematoxylin and eosin staining of the cortex (A) and olfactory bulb (B) regions of control non-infected (NF) and WNV H8912-infected mouse (IF) brain sections at day 45 post- i.c. infection. (TIF) [file pntd.0002275.s002.tif]
